# Efficacy of Docetaxel Plus Ramucirumab for Malignant Pleural Effusion and Cerebral Edema in Patients With Advanced Non‐Small Cell Lung Cancer: A Single‐Institution Retrospective Study

**DOI:** 10.1111/1759-7714.70295

**Published:** 2026-05-01

**Authors:** Meiko Morita, Kazushige Wakuda, Suguru Matsuda, Motoki Sekikawa, Keita Miura, Hiroaki Kodama, Michitoshi Yabe, Nobuaki Mamesaya, Haruki Kobayashi, Ryo Ko, Akira Ono, Hirotsugu Kenmotsu, Tateaki Naito, Haruyasu Murakami, Toshiaki Takahashi

**Affiliations:** ^1^ Division of Thoracic Oncology Shizuoka Cancer Center Shizuoka Japan

**Keywords:** cerebral edema, docetaxel, malignant pleural effusion, non‐small cell lung cancer, ramucirumab

## Abstract

**Background:**

Malignant pleural effusion (MPE) is associated with a poor prognosis and quality of life in patients with non‐small cell lung cancer (NSCLC). Additionally, cerebral edema can lead to neurological symptoms that adversely affect activities of daily living. While bevacizumab has demonstrated efficacy in treating both MPE and cerebral edema, there is limited research on ramucirumab, an angiogenesis inhibitor. Therefore, this study aimed to evaluate the efficacy of docetaxel combined with ramucirumab for the management of MPE and cerebral edema.

**Methods:**

We retrospectively analyzed medical records of patients with advanced NSCLC who received docetaxel in conjunction with ramucirumab at Shizuoka Cancer Center between August 2016 and March 2023. The primary endpoints were pleural effusion progression‐free survival (PE‐PFS) and the cerebral edema control rate. Secondary endpoints included response rate, progression‐free survival (PFS), overall survival (OS), and the incidence of toxicities.

**Results:**

A total of 163 patients were included. The median PE‐PFS was 8.1 months (95% CI: 4.8–12.0 months). The pleural effusion control rate was 87%, whereas the cerebral edema control rate was 26%. The response rate was 26%, with a median PFS of 4.4 months (95% confidence interval [CI]: 3.7–5.1 months) and a median OS of 11.1 months (95% CI: 9.2–16.0 months). Adverse events leading to discontinuation of treatment occurred in 30% of patients for docetaxel and 33% for ramucirumab, with fatigue being the most common reason for discontinuation.

**Conclusion:**

Docetaxel plus ramucirumab was effective in controlling pleural effusion but showed limited effects on cerebral edema.

## Graphical abstract

Docetaxel plus ramucirumab achieved effective control of malignant pleural effusion but showed modest benefit for cerebral edema in patients with advanced non‐small cell lung cancer.

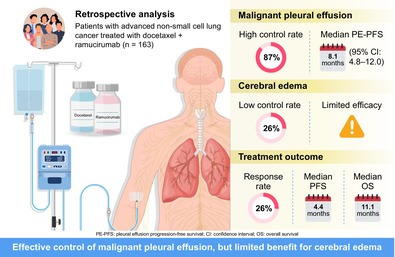

## 1 Introduction

Malignant pleural effusion (MPE) occurs in approximately 15%–20% of patients with advanced non‐small cell lung cancer (NSCLC) and is associated with poor prognosis and impairs quality of life [1]. Intrapleural therapy, utilizing a chest tube, is commonly employed to manage pleural effusion, with pleurodesis using agents such as talc, OK‐432, or bleomycin. A meta‐analysis by Dipper et al. [2] indicated that talc is associated with a lower failure rate in this context. However, prospective studies assessing intrapleural therapy with talc or bleomycin have reported success rates ranging from 37% to 84% within 3 months post‐treatment initiation, highlighting the variability in efficacy [3, 4, 5, 6, 7, 8]. Recent randomized trials comparing different talc pleurodesis techniques have further refined local management strategies for MPE [9]. Additionally, among patients who underwent immune checkpoint inhibitor (ICI) therapy following talc pleurodesis, interstitial lung disease (ILD) was observed in 32.7% of cases, suggesting that such complications may influence subsequent treatment strategies [10]. Furthermore, the overall response rate for MPE recurrence in patients treated with tyrosine kinase inhibitors has been reported to be 65.5%, indicating that these therapeutic agents may also effectively manage pleural effusion [11].

Common metastatic sites for NSCLC include the lungs, brain, bones, liver, and adrenal glands, with brain metastases occurring in approximately 25%–30% of patients [12]. Cerebral edema may be present at diagnosis or develop as an adverse effect of radiation therapy. In cases of radiation‐induced cerebral edema, quality of life can be significantly compromised even when the primary tumor is well controlled [13]. Corticosteroids are frequently utilized to manage cerebral edema; however, prolonged use may lead to various adverse effects [14, 15]. Bevacizumab, an anti–vascular endothelial growth factor (VEGF) antibody and angiogenesis inhibitor, has demonstrated antitumor efficacy in patients with NSCLC and MPE [16, 17]. It has also been reported to be effective in treating symptomatic brain radiation necrosis [13].

Ramucirumab, like bevacizumab, is an angiogenesis inhibitor but functions as a monoclonal antibody targeting VEGF receptor 2 (VEGFR‐2). Its efficacy has been established in the REVEL trial; however, there are limited reports regarding its effectiveness in patients with MPE or cerebral edema [18, 19]. Therefore, this study aimed to evaluate the efficacy of docetaxel combined with ramucirumab in controlling MPE and cerebral edema, as well as to assess its response rate and survival outcomes.

## 2 Methods

### 2.1 Study Population

The medical records of 163 patients with NSCLC who received docetaxel combined with ramucirumab therapy at Shizuoka Cancer Center between August 2016 and March 2023 were retrospectively reviewed. The data cut‐off date was established as August 31, 2023. Histological and cytological diagnoses were conducted in accordance with the World Health Organization classification criteria [20]. All patients were staged based on the seventh edition of the Tumor, Node, Metastasis classification by the International Association for the Study of Lung Cancer [21]. Antitumor efficacy was evaluated according to the Response Evaluation Criteria in Solid Tumors version 1.1, while adverse events were assessed using the Common Terminology Criteria for Adverse Events version 5.0 [22].

This study received approval from the Institutional Review Board of Shizuoka Cancer Center (IRB registration number: J2024‐183). Informed consent from patients was obtained using the opt‐out method.

### 2.2 Clinical Assessment

MPE was defined as an effusion confirmed through cytological analysis. Re‐accumulation of MPE was identified as a clear increase compared to baseline findings on chest radiography or computed tomography (CT). Pleural effusion progression‐free survival (PE‐PFS) was defined as the duration from the initiation of docetaxel plus ramucirumab therapy to the date of re‐accumulation of MPE, regardless of progression in other lesions, or to the date of death from any cause, whichever occurred first. The MPE control rate was calculated as the percentage of patients who did not experience re‐accumulation of MPE, characterized by an unequivocal increase compared to baseline on chest radiography or CT. Patients without re‐accumulation of MPE who experienced progression of other lesions and subsequently transitioned to the next line of therapy were censored at the time of treatment transition due to progression of these lesions. The cerebral edema control rate was defined as the proportion of patients exhibiting a reduction in high‐signal areas indicative of cerebral edema compared to baseline on T2‐weighted or fluid‐attenuated inversion recovery images from brain magnetic resonance imaging (MRI). The evaluation of pleural effusion was generally performed using chest radiography or computed tomography at intervals of approximately 6–8 weeks in routine clinical practice. Brain imaging was performed using MRI or CT according to the clinical indication, typically every 2–3 months during follow‐up. Cerebral edema was defined as peritumoral edema surrounding brain metastases confirmed on radiological imaging, including MRI or CT. Thoracentesis or pleural drainage was performed before the initiation of docetaxel plus ramucirumab therapy according to clinical indications.

### 2.3 Statistical Analysis

Progression‐free survival (PFS) was defined as the interval from the first administration of docetaxel plus ramucirumab to disease progression, death from any cause, or the end of follow‐up. Overall survival (OS) was defined as the duration from the first administration of docetaxel plus ramucirumab to death.

Survival probabilities were estimated using the Kaplan–Meier method, with a *p*‐value < 0.05 deemed statistically significant. All analyses were conducted using EZR version 1.60 (Jichi Medical University, Saitama Medical Center, Saitama, Japan) [23].

## 3 Results

### 3.1 Patient Characteristics

**FIGURE 1:**
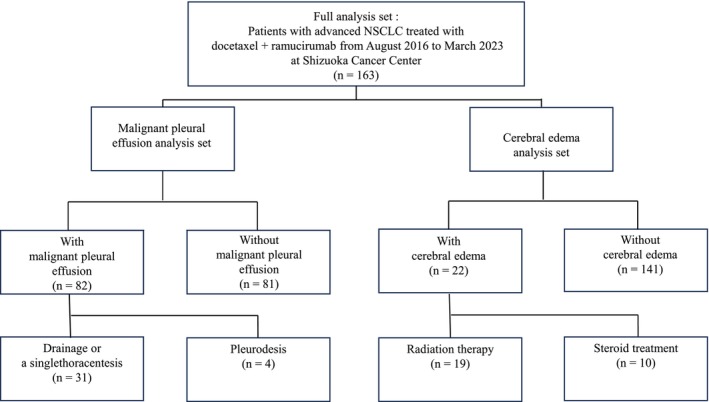
Flow diagram illustrating cohort classification prior to the initiation of docetaxel plus ramucirumab therapy. Patients were categorized according to the presence of malignant pleural effusion (MPE) and cerebral edema. Some patients received both drainage and pleurodesis, while others did not undergo pleural intervention. Patients could be included in both the MPE and cerebral edema analysis sets. NSCLC, non‐small cell lung cancer.

A flow diagram illustrating patient enrollment for this analysis is presented in Figure 1. A total of 163 patients constituted the full analysis set. Among them, 82 patients had malignant pleural effusion and were included in the MPE analysis, while 22 patients had cerebral edema and were included in the cerebral edema analysis. The characteristics of the 163 patients are summarized in Table 1. The median age at the commencement of docetaxel plus ramucirumab therapy was 66 years (range, 34–80 years), and 65.6% (*n* = 107) of the patients were male. The Eastern Cooperative Oncology Group Performance Status was 0 in 19% of patients, 1 in 78.5%, and 2 in 2.5%. A smoking history was observed in 75.5% of patients, while adenocarcinoma constituted 83.4% of the cohort. Clinical stage IV disease was present in 75.5% of patients, whereas 24.5% had recurrent disease. *EGFR* gene mutations were identified in 33.7% of patients, negative in 62.6%, and unknown in 3.7%. *ALK* fusion gene mutations were positive in 3.7%, negative in 77.3%, and unknown in 19%. The baseline characteristics of patients in the MPE and cerebral edema groups were generally comparable to those of the overall cohort, with no clinically significant imbalances noted between the groups.

**TABLE 1:** Baseline patient characteristics of the overall cohort, the malignant pleural effusion (MPE) group, and the cerebral edema group.

	All (*N* = 163)	MPE group (*N* = 82)	Cerebral edema group (*N* = 22)
	*N* (%)	*N* (%)	*N* (%)
Age			
Median	66	67	62
Range	34–80	40–80	42–76
Sex			
Male	107 (65.6)	55 (67.1)	15 (68.2)
Female	56 (34.4)	27 (32.9)	7 (31.8)
ECOG PS			
0	31 (19.0)	11 (13.4)	2 (9.0)
1	128 (78.5)	69 (84.1)	20 (91.0)
2	4 (2.5)	2 (2.4)	0 (0.0)
Smoking status			
Ever	123 (75.5)	61 (74.4)	4 (18.2)
Never	40 (24.5)	21 (25.6)	18 (81.8)
Histology			
Adenocarcinoma	136 (83.4)	68 (82.9)	18 (81.8)
Other	27 (16.6)	14 (17.1)	4 (18.2)
Clinical stage			
Stage IV	123 (75.5)	59 (72.0)	19 (86.4)
Other	40 (24.5)	23 (28.0)	3 (13.6)
EGFR mutant			
Positive	55 (33.7)	32 (39.0)	7 (31.8)
Negative	102 (62.6)	46 (56.1)	15 (68.2)
Unknown	6 (3.7)	4 (4.9)	0 (0.0)
ALK fusion			
Positive	6 (3.7)	3 (3.7)	0 (0.0)
Negative	126 (77.3)	60 (73.2)	20 (91.0)
Unknown	31 (19.0)	19 (23.1)	2 (9.0)
Treatment line of docetaxel plus ramucirumab			
2	73 (44.8)	36 (43.9)	9 (41.0)
≥ 3	90 (55.2)	46 (56.1)	13 (59.0)

*Note:* Data are presented as *n* (%) unless otherwise indicated. Age is presented as median (range).

Abbreviations: ALK, anaplastic lymphoma kinase; ECOG PS, Eastern Cooperative Oncology Group performance status; EGFR, epidermal growth factor receptor; MPE, malignant pleural effusion.

### 3.2 The Efficacy of Docetaxel Plus Ramucirumab

**FIGURE 2:**
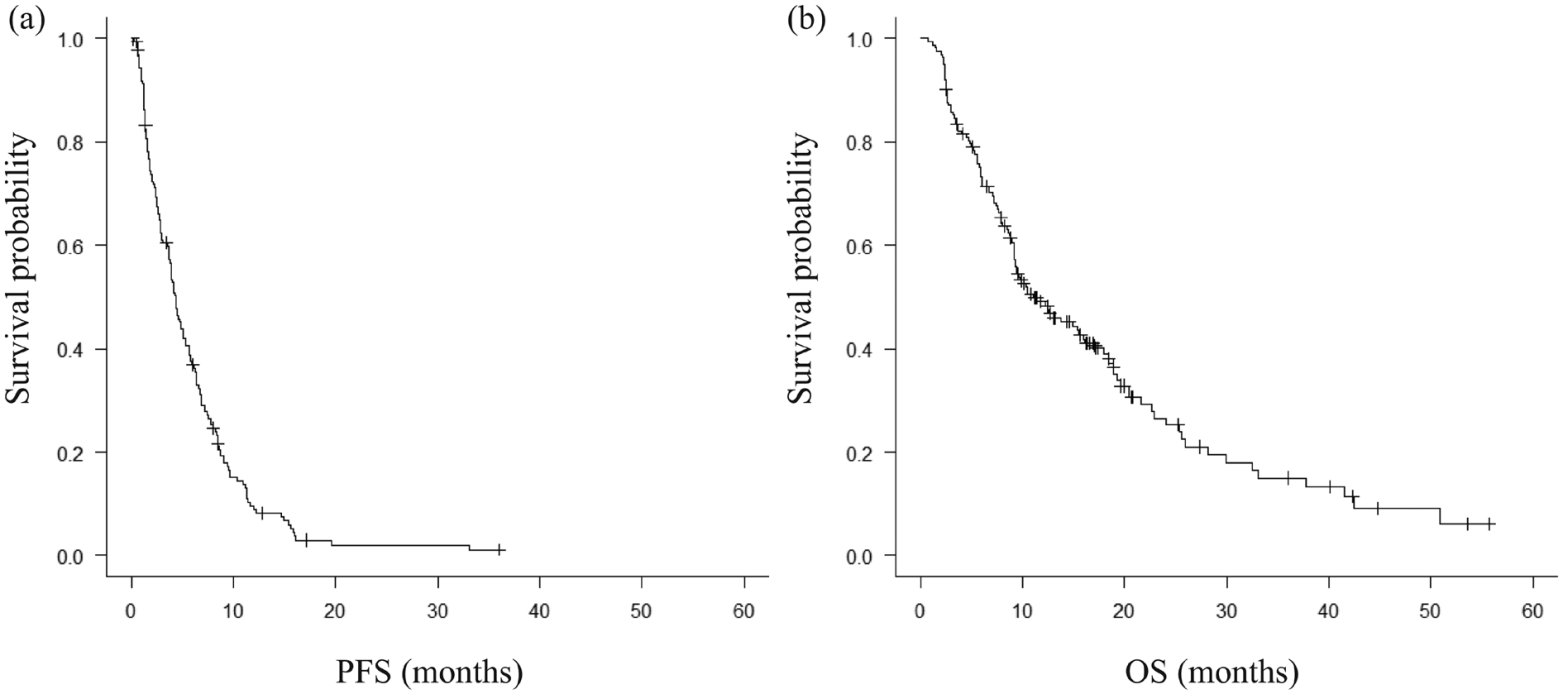
Kaplan–Meier curves depicting progression‐free survival (PFS) and overall survival (OS) within the entire study population. (A) PFS. (B) OS.

The median observation period was 15.4 months (range, 2.6–56.5 months). The median number of treatment cycles was 4 (range, 1–21) for both docetaxel and ramucirumab. The response rate was 26%, with a median PFS of 4.4 months (95% confidence interval [CI]: 3.7–5.1 months) and a median OS of 11.1 months (95% CI: 9.2–16.0 months; Table 2; Figure 2). Subgroup analyses based on histological subtype and *EGFR* mutation status revealed no significant differences in either PFS or OS. Disease progression was observed in 101 patients during treatment with docetaxel plus ramucirumab. Among these patients, 73 proceeded to subsequent systemic therapy, while the remaining patients received best supportive care.

**TABLE 2:** Treatment efficacy outcomes of docetaxel plus ramucirumab in the overall study population.

		*N* = 163	
		*N*	%
Number of treatment cycles	Median	4	
	Range	1–21	
Best response	CR	1	0.6%
	PR	41	25.2%
	SD	79	48.5%
	PD	33	20.2%
	Non CR/Non PD	1	0.6%
	NE	8	4.9%

Abbreviations: CI, confidence interval; OS, overall survival; PFS, progression‐free survival.

### 3.3 MPE

**FIGURE 3:**
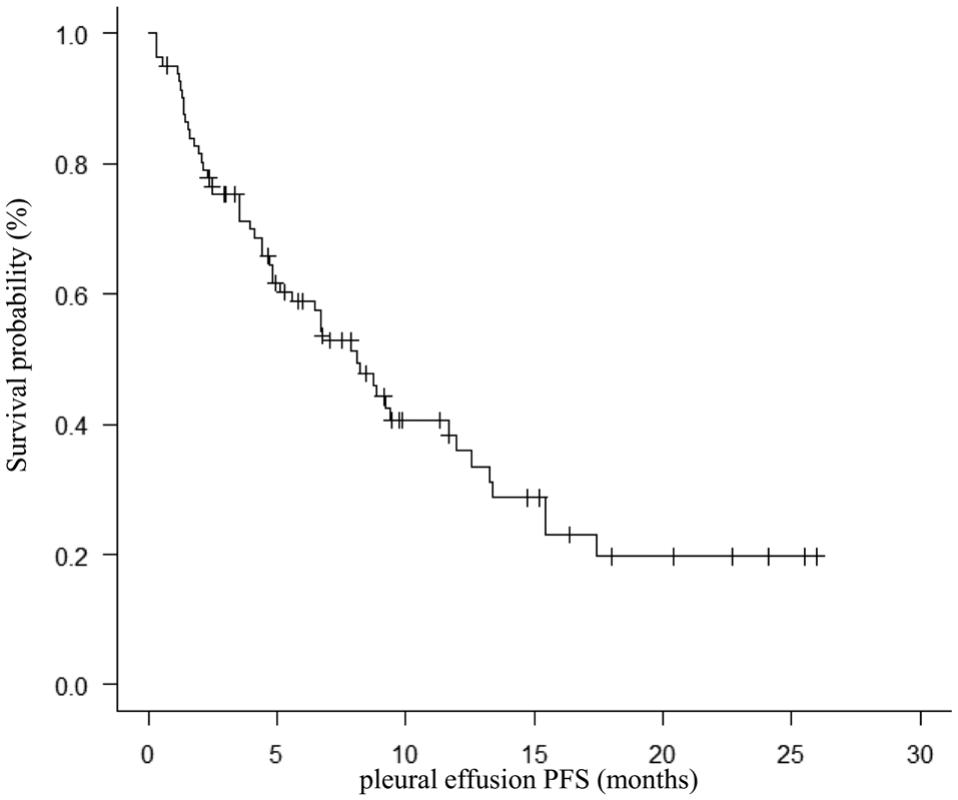
Kaplan–Meier curve illustrating pleural effusion progression‐free survival (PE‐PFS) in patients with malignant pleural effusion.

**FIGURE 4:**
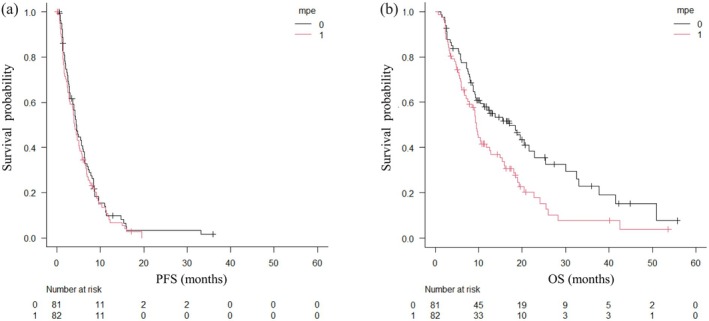
Kaplan–Meier curves demonstrating survival outcomes according to the presence or absence of malignant pleural effusion (MPE). (A) Progression‐free survival (PFS). (B) Overall survival (OS). MPE: 0 = absence, 1 = presence.

Prior to the initiation of docetaxel plus ramucirumab therapy, MPE was observed in 82 patients (50%). Drainage or a single thoracentesis was performed in 31 patients (19%), and pleurodesis using talc was conducted in 4 patients (2%). The median PE‐PFS was 8.1 months (95% CI: 4.8–12.0 months; Figure 3). The MPE control rate at the time of best response was 87%. Kaplan–Meier analysis demonstrated that the median PFS was 4.0 months (95% CI: 2.9–5.1 months) in the MPE group and 4.4 months (95% CI: 3.1–6.1 months) in the group without MPE (log‐rank *p* = 0.523; Figure 4A). The median OS was 9.4 months (95% CI: 7.5–12.5 months) in the MPE group, compared to 17.1 months in the group without MPE (log‐rank *p* = 0.009; Figure 4B). Among patients with MPE, the control rate was 92% (46/50) in patients with prior ICI and 78% (25/32) in those without prior ICI therapy. Subgroup analyses based on histological subtype and *EGFR* mutation status showed no significant differences in PE‐PFS or MPE control rate.

### 3.4 Cerebral Edema

**FIGURE 5:**
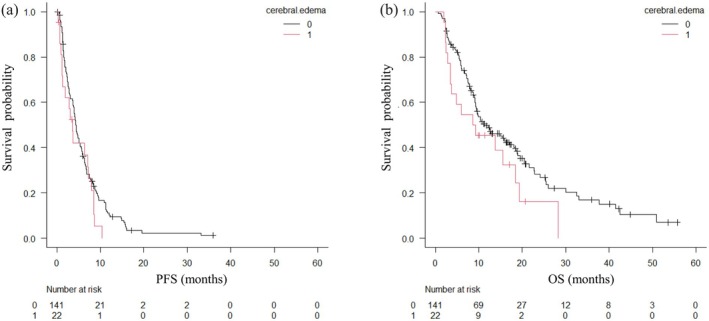
Kaplan–Meier curves of survival outcomes according to the presence or absence of cerebral edema. (A) Progression‐free survival (PFS). (B) Overall survival (OS). Cerebral edema: 0 = absence, 1 = presence.

At the initiation of docetaxel plus ramucirumab therapy, cerebral edema was observed in 22 patients (13%). The cerebral edema control rate was 26%. Among these patients, 19 (86%) had previously undergone radiation therapy to the brain. Steroid therapy had been initiated prior to docetaxel plus ramucirumab in 10 patients (45%), including those who continued steroid treatment at the time of treatment initiation. The median maximum diameter of brain metastases was 9.7 mm (95% CI: 6.6–28.8 mm), and the disease control rate for brain metastases was 41%. The median follow‐up interval for MRI assessments was 84 days (95% CI: 63–92 days). Kaplan–Meier analyses showed no significant difference in progression‐free survival between patients with and without cerebral edema (log‐rank *p* = 0.149; Figure 5A). There was also no apparent difference in overall survival between the two groups (Figure 5B). Subgroup analyses based on histological subtype and *EGFR* mutation status revealed no significant differences in the cerebral edema control rate.

### 3.5 Safety

Table 3 outlines the adverse events recorded during this study. Primary prophylactic granulocyte‐colony stimulating factor (G‐CSF) was administered to 81% of patients, while secondary prophylactic G‐CSF was administered to 1%. Grade 3 or higher neutropenia was identified in 32 patients (20%), while febrile neutropenia (FN) of Grade 3 or higher was noted in 4 patients (3%). Proteinuria was reported in 34 patients (21%) across all grades, with Grade 3 or higher observed in 1 patient (1%). Hypertension was documented in 21 patients (13%), including 1 patient (1%) with Grade 3 or higher hypertension. Hemorrhage occurred in 40 patients (25%), with 2 patients (1%) experiencing Grade 3 or higher. Limb edema was reported in 47 patients (29%), although no cases of Grade 3 or higher were noted.

**TABLE 3:** Treatment‐related adverse events according to CTCAE version 5.0.

	*N* = 163			
	Any Gr		≧ Gr3	
	*N*	%	*N*	%
Anemia	43	26.4	5	3.1
White blood cell count decreased	38	23.3	20	12.3
Neutrophil count decreased	38	23.3	32	19.6
Platelet count decreased	34	20.9	5	3.1
Febrile neutropenia	4	2.5	4	2.5
Edema limbs	47	28.8	0	0
Hemorrhage	40	24.5	2	1.2
Proteinuria	34	20.9	1	0.6
Hypertension	21	12.9	1	0.6
Pleural effusion	12	7.4	0	0
Pneumonitis	8	4.9	5	3.1
Infusion reaction	3	1.8	2	1.2

*Note:* Data are presented as *n* (%).

Abbreviations: CTCAE, Common Terminology Criteria for Adverse Events; FN, febrile neutropenia.

Table 4 details the treatment courses. Regarding docetaxel, dose interruption was observed in 3 patients (2%), dose reduction in 40 patients (25%), and treatment discontinuation in 49 patients (30%). For ramucirumab, the corresponding numbers were 12 patients (7%) for dose interruption, 11 patients (6%) for dose reduction, and 53 patients (33%) for treatment discontinuation. The primary reason for discontinuation of docetaxel was fatigue, affecting 15 patients (31%), followed by limb edema in 10 patients (20%) and anorexia in 7 patients (14%). For ramucirumab, the leading cause of discontinuation was also fatigue, reported in 13 patients (25%), followed by anorexia in 7 patients (13%) and hemorrhage in 6 patients (11%).

**TABLE 4:** Treatment exposure and reasons for dose modification or discontinuation of docetaxel and ramucirumab.

	*N* = 163			
	Docetaxel		Ramucirumab	
	*N*	%	*N*	%
Treatment cessation	3	1.8	12	7.4
Dose reduction	40	24.5	11	6.7
Treatment discontinuation	49	30.1	53	32.5
Reasons: Fatigue	15	30.6	13	24.5
Edema limbs	10	20.4	4	7.5
Anorexia	7	14.3	7	13.2

*Note:* Data are presented as *n* (%).

Relative dose intensity was not formally evaluated in this retrospective study because treatment schedules and dose modifications were determined individually according to the clinical condition of each patient.

## 4 Discussion

The results of this study evaluating the efficacy of docetaxel plus ramucirumab therapy indicated a median PFS of 4.4 months (95% CI: 3.7–5.1 months) and a median OS of 11.1 months (95% CI: 9.2–16.0 months), which aligns with previously reported data [18, 24].

The Japanese randomized phase II trial (JVCG trial) reported significant hematologic toxicities, including neutropenia in 94.7% of patients, with Grade 3 or higher in 89.5%, and FN of Grade 3 or higher in 34.2% [23]. In contrast, our study observed neutropenia in 23% of patients, with Grade 3 or higher in 20%, and FN in 3%, indicating a lower incidence compared to the JVCG trial. This discrepancy may be attributed to the high use of prophylactic G‐CSF in our study, with primary prophylaxis administered to 81% of patients and secondary prophylaxis to 1%. Other non‐hematologic toxicities observed in our study were consistent with previously reported data [18, 24].

Concerning the efficacy of angiogenesis inhibitors for MPE, two phase II trials have been conducted involving bevacizumab. The first was a single‐arm phase II trial focusing on chemotherapy‐naïve patients treated with carboplatin, paclitaxel, and bevacizumab for advanced non‐squamous NSCLC with MPE [16]. In that study, MPE was assessed biweekly using chest radiography and physical examinations, resulting in an MPE control rate of 91.8% [15]. The second trial was also a single‐arm phase II trial and involved a similar patient population treated with carboplatin, pemetrexed, and bevacizumab. The MPE control rate calculated for patients not requiring pleurodesis after 8 weeks of treatment was 93% (95% CI: 77%–99%) [17]. For ramucirumab, a single‐arm phase II trial has reported that in patients with NSCLC and MPE who had previously undergone platinum‐based chemotherapy, the MPE control rate reached 100% at 8 weeks following the initiation of docetaxel plus ramucirumab therapy [19]. In the present study, pleural effusion control was evaluated using PE‐PFS rather than a fixed time‐point assessment such as an 8‐week control rate. In our study, the MPE control rate at the time of best response was 87%, suggesting that ramucirumab is also an effective treatment option for MPE in real‐world clinical practice. However, due to the single‐arm design of our study, it is challenging to isolate the anti‐angiogenic effects of ramucirumab from the additive antitumor effects of docetaxel.

One randomized trial has been conducted on the impact of angiogenesis inhibitors on cerebral edema. This trial involved patients with symptomatic radiation brain necrosis, comparing bevacizumab with a placebo, administered every 3 weeks for 2 cycles. MRI findings and neurological symptoms were assessed 3 weeks after the second administration [25]. The results demonstrated that all patients in the bevacizumab group exhibited improvements on MRI, whereas no responses were noted in the placebo group [25]. Similarly, neurological improvement was observed in all patients receiving bevacizumab, while no such improvement was noted in the placebo group [25]. To date, no studies have evaluated the effects of ramucirumab in comparable settings. In our study, the cerebral edema control rate was 26%, which is lower than the previously reported rate for bevacizumab. This finding suggests that, despite both bevacizumab and ramucirumab being classified as angiogenesis inhibitors, their therapeutic effects on brain edema may differ.

Two angiogenesis inhibitors, bevacizumab and ramucirumab, are utilized in the treatment of lung cancer. Bevacizumab is a monoclonal antibody that binds to VEGF‐A, inhibiting not only the VEGF‐A/VEGFR‐2 pathway but also the VEGF‐A/VEGFR‐1 and NRP‐1/2 pathways [26]. In contrast, ramucirumab is a monoclonal antibody that specifically binds to VEGFR‐2, thereby blocking the VEGFR‐2 pathways associated with VEGF‐A, VEGF‐C, and VEGF‐D [26]. Although the expression and function of VEGF in the brain have been explored, the impact of the differing mechanisms of action between these two angiogenesis inhibitors on therapeutic efficacy remains unclear [27]. Understanding this discrepancy is essential for optimizing the application of angiogenesis inhibitors in lung cancer treatment. The limited effect of docetaxel plus ramucirumab on cerebral edema observed in this study may reflect the clinical background of the study population. Most patients had previously undergone brain radiotherapy, and approximately half of the patients required corticosteroid therapy at the time of treatment initiation, suggesting that cerebral edema may have already been difficult to control in some cases. These factors may have limited the potential impact of anti‐angiogenic therapy on cerebral edema.

This study has three limitations. First, it was conducted at a single institution, resulting in a small sample size; institution‐specific treatment policies and patient characteristics might have influenced the findings, thereby limiting their generalizability. Second, the retrospective design of the study could not fully eliminate selection bias. Specifically, patients with refractory cerebral edema or MPE may have been preferentially treated with docetaxel plus ramucirumab, potentially leading to channeling bias. This may have influenced the observed treatment outcomes in this retrospective analysis. Third, the definition of cerebral edema control was somewhat subjective, which may restrict the reproducibility of outcome assessments. Additionally, the heterogeneity in MRI follow‐up intervals could have impeded the accurate evaluation of cerebral edema, complicating the conclusion that the effects of ramucirumab on cerebral edema were limited. Predictive biomarkers for response to anti‐angiogenic therapy were not evaluated in this study. Although VEGF‐related pathways are known to be involved in the development of malignant pleural effusion, biomarkers predicting the response to angiogenesis inhibitors have not been well established. Further studies may help clarify potential predictors of response to ramucirumab in patients with MPE.

Future studies should prioritize prospective randomized trials that compare angiogenesis inhibitors, including ramucirumab and bevacizumab. Moreover, identifying predictive factors for the efficacy of ramucirumab may enhance strategies for selecting appropriate angiogenesis inhibitors. These findings may also have implications for real‐world clinical practice. In patients with advanced NSCLC complicated by malignant pleural effusion, docetaxel plus ramucirumab may represent a practical therapeutic option for controlling pleural effusion when treatment options are limited.

## 5 Conclusion

The combination of docetaxel and ramucirumab demonstrated a substantial control rate for MPE in patients with advanced NSCLC, indicating its clinical utility in real‐world practice. Conversely, the impact on cerebral edema appeared to be limited, which may differ from previously reported outcomes associated with bevacizumab. Docetaxel plus ramucirumab may represent a viable treatment option for patients with MPE. Further large‐scale prospective studies and direct comparisons with other angiogenesis inhibitors are warranted.

## Author Contributions

**Meiko Morita:** conceptualization, methodology, data curation, formal analysis, investigation, visualization, writing – original draft, writing – review and editing, project administration. **Kazushige Wakuda:** conceptualization, methodology, supervision, writing – review and editing. **Suguru Matsuda:** investigation, data curation, writing – review and editing. **Motoki Sekikawa:** investigation, writing – review and editing, data curation. **Keita Miura:** investigation, writing – review and editing, data curation. **Hiroaki Kodama:** investigation, writing – review and editing, data curation. **Michitoshi Yabe:** investigation, writing – review and editing, data curation. **Nobuaki Mamesaya:** investigation, writing – review and editing, data curation. **Haruki Kobayashi:** investigation, writing – review and editing, data curation. **Ryo Ko:** investigation, writing – review and editing, data curation. **Akira Ono:** investigation, writing – review and editing, data curation. **Hirotsugu Kenmotsu:** supervision, investigation, writing – review and editing. **Tateaki Naito:** investigation, writing – review and editing, supervision. **Haruyasu Murakami:** supervision, writing – review and editing, investigation. **Toshiaki Takahashi:** investigation, writing – review and editing, supervision.

## Funding

The authors have nothing to report.

## Conflicts of Interest

The authors declare no conflicts of interest.

## Data Availability

The data that support the findings of this study are available on request from the corresponding author. The data are not publicly available due to privacy or ethical restrictions.

## References

[1] P. C. Kulandaisamy , S. Kulandaisamy , D. Kramer , and C. McGrath , “Malignant Pleural Effusions—A Review of Current Guidelines and Practices,” Journal of Clinical Medicine 10, no. 23 (2021): 5535. 34884236 10.3390/jcm10235535 PMC8658426

[2] A. Dipper , H. E. Jones , R. Bhatnagar , N. J. Preston , N. Maskell , and A. O. Clive , “Interventions for the Management of Malignant Pleural Effusions: A Network Meta‐Analysis,” Cochrane Database of Systematic Reviews 4 (2020): CD010529. 32315458 10.1002/14651858.CD010529.pub3 PMC7173736

[3] A. Goodman and C. W. Davies , “Efficacy of Short‐Term Versus Long‐Term Chest Tube Drainage Following Talc Slurry Pleurodesis in Patients With Malignant Pleural Effusions: A Randomized Trial,” Lung Cancer 54 (2006): 51–55. 16920219 10.1016/j.lungcan.2006.06.004

[4] A. Stefani , P. Natali , C. Casali , and U. Morandi , “Talc Poudrage Versus Talc Slurry in the Treatment of Malignant Pleural Effusion. A Prospective Comparative Study,” European Journal of Cardio‐Thoracic Surgery 30 (2006): 827–832. 17113008 10.1016/j.ejcts.2006.10.002

[5] S. Kolschmann , A. Ballin , and A. Gillissen , “Clinical Efficacy and Safety of Thoracoscopic Talc Pleurodesis in Malignant Pleural Effusions,” Chest 128 (2005): 1431–1435. 16162739 10.1378/chest.128.3.1431

[6] C. M. Dresler , J. Olak , J. E. Herndon, II , et al., “Phase III Intergroup Study of Talc Poudrage Versus Talc Slurry Sclerosis for Malignant Pleural Effusion,” Chest 127 (2005): 909–915. 15764775 10.1378/chest.127.3.909 PMC4644736

[7] M. Paschoalini , F. S. Vargas , E. Marchi , et al., “Prospective Randomized Trial of Silver Nitrate vs Talc Slurry in Pleurodesis for Symptomatic Malignant Pleural Effusions,” Chest 128 (2005): 684–689. 16100154 10.1378/chest.128.2.684

[8] S. Sartori , D. Tassinari , P. Ceccotti , et al., “Prospective Randomized Trial of Intrapleural Bleomycin Versus Interferon Alfa‐2b via Ultrasound‐Guided Small‐Bore Chest Tube in the Palliative Treatment of Malignant Pleural Effusions,” Journal of Clinical Oncology 22 (2004): 1228–1233. 15051770 10.1200/JCO.2004.09.164

[9] R. Bhatnagar , H. E. G. Piotrowska , M. Laskawiec‐Szkonter , et al., “Effect of Thoracoscopic Talc Poudrage vs Talc Slurry via Chest Tube on Pleurodesis Failure Rate Among Patients With Malignant Pleural Effusions: A Randomized Clinical Trial,” Journal of the American Medical Association 323 (2020): 60–69. 31804680 10.1001/jama.2019.19997 PMC6990658

[10] S. Tanaka , K. Nozaki , S. Watanabe , et al., “Risk of Lung Injury With Immune Checkpoint Inhibitors After Talc Pleurodesis: A Retrospective Study,” Lung Cancer 204 (2025): 108590. 40412103 10.1016/j.lungcan.2025.108590

[11] W. Wang , Q. Zhou , K. Chen , et al., “Intracavitary Chemotherapy With EGFR‐TKI is Not Superior to TKI Monotherapy in Controlling Malignant Pleural Effusion Recurrence in EGFR‐Mutated Non‐Small Cell Lung Cancer,” Journal of Thoracic Disease 11 (2019): 4196–4204. 10.21037/jtd.2019.09.36 PMC6790470 31656643

[12] C. J. Langer and M. P. Mehta , “Current Management of Brain Metastases, With a Focus on Systemic Options,” Journal of Clinical Oncology 23 (2005): 6207–6219. 16135488 10.1200/JCO.2005.03.145

[13] M. Furuse , N. Nonoguchi , T. Kuroiwa , et al., “A Prospective, Multicentre, Single‐Arm Clinical Trial of Bevacizumab for Patients With Surgically Untreatable, Symptomatic Brain Radiation Necrosis,” Neuro‐Oncology Practice 3 (2016): 272–280. 27833757 10.1093/nop/npv064 PMC5099992

[14] T. C. Ryken , M. McDermott , P. D. Robinson , et al., “The Role of Steroids in the Management of Brain Metastases: A Systematic Review and Evidence‐Based Clinical Practice Guideline,” Journal of Neuro‐Oncology 96 (2010): 103–114. 19957014 10.1007/s11060-009-0057-4 PMC2808527

[15] J. Drappatz , D. Schiff , S. Kesari , A. D. Norden , and P. Y. Wen , “Medical Management of Brain Tumor Patients,” Neurologic Clinics 25 (2007): 1035–1071. 17964025 10.1016/j.ncl.2007.07.015

[16] M. Tamiya , A. Tamiya , T. Yamadori , et al., “Phase 2 Study of Bevacizumab With Carboplatin‐Paclitaxel for Non‐Small Cell Lung Cancer With Malignant Pleural Effusion,” Medical Oncology 30 (2013): 676. 23925664 10.1007/s12032-013-0676-7

[17] K. Usui , S. Sugawara , M. Nishitsuji , et al., “A Phase II Study of Bevacizumab With Carboplatin‐Pemetrexed in Non‐Squamous Non‐Small Cell Lung Carcinoma Patients With Malignant Pleural Effusions: North East Japan Study Group Trial NEJ013A,” Lung Cancer 99 (2016): 131–136. 27565928 10.1016/j.lungcan.2016.07.003

[18] E. B. Garon , T. E. Ciuleanu , O. Arrieta , et al., “Ramucirumab Plus Docetaxel Versus Placebo Plus Docetaxel for Second‐Line Treatment of Stage IV Non‐Small‐Cell Lung Cancer After Disease Progression on Platinum‐Based Therapy (REVEL): A Multicentre, Double‐Blind, Randomised Phase 3 Trial,” Lancet 384 (2014): 665–673. 24933332 10.1016/S0140-6736(14)60845-X

[19] S. Takemoto , M. Fukuda , R. Ogata , et al., “Phase II Study of Ramucirumab and Docetaxel for Previously Platinum‐Treated Patients With Non‐Small Cell Lung Cancer and Malignant Pleural Effusion (PLEURAM Study),” Translational Lung Cancer Research 13 (2024): 2673–2682. 39507035 10.21037/tlcr-24-508 PMC11535820

[20] W. D. Travis , E. Brambilla , H. K. Müller‐Hermelink , and C. C. Harris , WHO Classification of Tumours of the Lung, Pleura, Thymus and Heart (IARC Press, 2004).

[21] P. Goldstraw , J. Crowley , K. Chansky , et al., “The IASLC Lung Cancer Staging Project: Proposals for the Revision of the TNM Stage Groupings in the Forthcoming (Seventh) Edition of the TNM Classification of Malignant Tumours,” Journal of Thoracic Oncology 2 (2007): 706–714. 17762336 10.1097/JTO.0b013e31812f3c1a

[22] E. A. Eisenhauer , P. Therasse , J. Bogaerts , et al., “New Response Evaluation Criteria in Solid Tumours: Revised RECIST Guideline (Version 1.1),” European Journal of Cancer 45 (2009): 228–247. 19097774 10.1016/j.ejca.2008.10.026

[23] Y. Kanda , “Investigation of the Freely Available Easy‐To‐Use Software ‘EZR’ for Medical Statistics,” Bone Marrow Transplantation 48 (2013): 452–458. 23208313 10.1038/bmt.2012.244 PMC3590441

[24] K. Yoh , Y. Hosomi , K. Kasahara , et al., “A Randomized, Double‐Blind, Phase II Study of Ramucirumab Plus Docetaxel vs Placebo Plus Docetaxel in Japanese Patients With Stage IV Non‐Small Cell Lung Cancer After Disease Progression on Platinum‐Based Therapy,” Lung Cancer 99 (2016): 186–193. 27565938 10.1016/j.lungcan.2016.07.019

[25] V. A. Levin , L. Bidaut , P. Hou , et al., “Randomized Double‐Blind Placebo‐Controlled Trial of Bevacizumab Therapy for Radiation Necrosis of the Central Nervous System,” International Journal of Radiation Oncology, Biology, Physics 79 (2011): 1487–1495. 20399573 10.1016/j.ijrobp.2009.12.061 PMC2908725

[26] L. M. Ellis and D. J. Hicklin , “VEGF‐Targeted Therapy: Mechanisms of Anti‐Tumour Activity,” Nature Reviews. Cancer 8 (2008): 579–591. 18596824 10.1038/nrc2403

[27] N. Ferrara , “VEGF and the Nervous System,” Nature Reviews. Neuroscience 5 (2004): 209–221.14976520

